# Relationship between behavioral and mood responses to monetary rewards in a sample of Indian students with and without reported pain

**DOI:** 10.1038/s41598-022-24821-2

**Published:** 2022-11-24

**Authors:** Tanya Tandon, Mayron Piccolo, Katharina Ledermann, Rashmi Gupta, Naser Morina, Chantal Martin-Soelch

**Affiliations:** 1 Unit of Clinical and Health Psychology, University of Fribourg, Fribourg, Switzerland; 2 Unit of Clinical and Health Psychology, University of Fribourg, Fribourg, Switzerland; 3 Department of Psychology, Harvard University, Cambridge, USA; 4 Department of Consultation-Liaison-Psychiatry, University Hospital Zurich, University of Zurich, Zurich, Switzerland; 5 Cognitive and Behavioural Neuroscience Laboratory, Department of Humanities and Social Sciences, Indian Institute of Technology Bombay, Mumbai, India

**Keywords:** Psychology, Pain

## Abstract

Physical pain has become a major health problem with many university students affected by it worldwide each year. Several studies have examined the prevalence of pain-related impairments in reward processing in Western, Educated, Industrialized, Rich, and Democratic (WEIRD) countries but none of the studies have replicated these findings in a non-western cultural setting. Here, we aimed to investigate the prevalence of physical pain symptoms in a sample of university students in India and replicate our previous study conducted on university students in Switzerland, which showed reduced mood and behavioral responses to reward in students with significant pain symptoms. We grouped students into a sub-clinical (N = 40) and a control group (N = 48) to test the association between pain symptoms and reward processes. We used the Fribourg reward task and the pain sub-scale of the Symptom Checklist (SCL-27-plus) to assess physical symptoms of pain. We found that 45% of the students reported high levels of physical symptoms of pain and interestingly, our ANOVA results did not show any significant interaction between reward and the groups either for mood scores or for outcomes related to performance. These results might yield the first insights that pain-related impairment is not a universal phenomenon and can vary across cultures.

## Introduction

Pain and reward have been shown to interact^1,2^. More specifically, several studies report the effects of chronic and acute pain on the neural processing of the reward^3,4^. For instance, a study conducted on twenty-eight patients with chronic pain showed pain-related alterations in the brain regions (i.e., reduced striatal activation) involved in reward processing while performing the Monetary Incentive Delay (MID) task as compared to healthy controls^5^. Similarly, chronic pain is highly comorbid with mood disorders^5,6^ and several studies report the presence of dysfunctional reward pathways in co-occurring pain and mood alteration^7,8^. For instance, winning rewards such as monetary rewards are associated with an increase in self-reported mood in healthy controls^9–11^ whereas impaired mood responsivity has been shown in people suffering from chronic pain^12^. This suggests that reward processing could be a mechanism underlying the relationship between pain and mood disorders^13^ and an increase in mood responses has been associated with neural changes in regions involved in reward processes^14^.

Pain also alters reward responsivity in non-chronic samples^11,15^. Based on the findings of pain-related impairments in reward processing, we tested whether pain-related impairment in reward processing could also be observed in a sub-clinical population i.e., university students. Our results showed that 49% of the students (*n* = 79) reported high levels of physical symptoms of pain. Moreover, students reporting non-chronic yet significant sub-clinical physical pain symptoms showed reduced mood responses to monetary reward (i.e., lower mood scores) when compared to students without sub-clinical pain symptoms^11^.

Physical pain has become a major health problem among university students, with around 54% of them being affected by it each year worldwide^16^. In western countries, for instance, in Switzerland, physical pain is the most disabling disease^17^, and over 80% of the students report lower back pain and neck pain yearly according to the Swiss Health Survey data^17,18^. In the UK, the prevalence of pain is 66.9% among students^19^. In the Netherlands, the 12-month prevalence was about 31.4% (neck pain), 30.3% (shoulder pain), and 17.5% (wrist/hand pain)^20^. Finally, in the USA, 81% of the students showed a high prevalence of musculoskeletal discomfort during/after computer use^21^. These studies have mainly been conducted in Western, Educated, Industrialized, Rich, and Democratic (WEIRD) countries and might not be representative of other cultural contexts which, on the other hand, include a large part of the world’s population^22^.

While several studies have examined the prevalence of physical pain symptoms in European countries and the United States^23,24^, only a few studies on physical pain in students in Asia specifically Southeast Asia^25^ were conducted, despite its high prevalence. This is important because, for instance, in India, physical pain is quite common among students, affecting 29%-81% of them each year^26^. Another cross-sectional study carried out in one of the universities in India (N = 160) showed that the prevalence of physical pain, especially lower back pain, was 45.3% among the students^27^. To our knowledge, none of the studies have shown an Indian perspective on the interaction between pain and reward processes though there seem to be differences in the experiences of pain in different cultures^28^. For instance, studies on the management of chronic chest pain showed that South Asian people waited twice as long as Europeans to visit a medical doctor after experiencing an onset of chest pain^29^. This has been suggested that it might be due to lower disease awareness and health-seeking behavior in South-Asian cultures than in European culture^30,31^ but further research is needed. Also, in many South Asian countries when compared to Western countries, individuals are encouraged to avoid pain even when they do experience it^32^ due to socio-economic factors such as lack of financial support or lack of access to health care^33^ since suffering from pain might lead to an increase in the financial burden of the families. Likewise, the perception of reward also differs across cultures, for example, a study by Jang, Shen, Allen, and Zhang^34^ showed cultural variations in the relationship between reward and motivation. For instance, in a country like Pakistan, which is characterized by a collectivist culture^35^ and predominantly masculine values^36^, monetary rewards were evaluated as highly attractive as masculine societies emphasize success based on material gain^37^ in such societies, individuals with high collectivist values demonstrate a greater preference for monetary benefits^38^. Finally, Chiang and Birtch^39^ found that employees in Hong Kong with collectivist values demonstrated a higher monetary reward orientation than Finnish employees with individualistic values. Due to these potential cultural differences in the perception of pain and reward, it is important to extend the current literature on this topic to non-western cultures.

Investigating the association between pain and reward processing is important since research has shown that reward processes and the motivation to achieve rewards play an important role in students learning processes in educational settings^40^. For instance, a meta-analysis by Cerasoli et al.^41^ showed that extrinsic incentives (e.g., such as monetary rewards) are the best predictors of performance in university students and the presence of extrinsic incentives encourages students to perform better. Therefore, if these reward processes are affected due to chronic or acute pain, it might lead, in turn, to poorer academic performance in students, higher absenteeism, and poorer peer and social functioning^42^ which are also important factors to experience reinforcing activities during student’s life.

Considering that few studies have focused on the prevalence of pain in students in India and the lack of studies investigating reward and pain interaction in non-WEIRD populations, the first aim of our study was to investigate the prevalence of physical pain symptoms in a sample of university students in India which although being educated, represents non-western cultural aspects. The second aim was to replicate our previous study conducted on university students in Switzerland^11^ in a non-western cultural context i.e., in India. We were interested to see the prevalence of physical pain symptoms in a sample of university students in India and hypothesized that participants with sub-clinical pain scores would display a reduction of the effect of monetary reward on mood when compared to the participants without any clinically significant physical pain symptoms. We hypothesized that there would be an effect of reward on mood and performance (i.e., with reduced reaction times and increased mood scores in response to reward) and that this effect would be reduced in participants with sub-clinical pain. To test that, we used the Fribourg Reward task that had successfully differentiated mood responses between sub-clinical and control samples in a previous study of our group performed in a sample of Swiss students^11^. Similar to^11^, students were categorized into a sub-clinical group, i.e., university students with non-chronic yet clinically significant pain symptoms, and a control group, i.e., university students without reported physical pain a posteriori and based on their self-reported pain scores. We compared the groups and investigated the effects of reward on mood and performance using the Fribourg reward task^10^ in each group to test the association between pain symptoms and reward processes in Indian students.

## Method

### Participants

Participants were recruited through flyers and emails from several Universities in India. General inclusion criteria were that students should be 18 years of age and have a good command of English. Table 1 provides the participants’ demographics. The sample comprised 88 students (*M*_age_ = 21.77 years, *SD* = 2.31; 50% Females). We used a cross-sectional design and assigned the participants a posteriori, i.e., the participants were categorized into two groups after the completion of the study: a sub-clinical pain group (N = 40) and a control group (N = 48) according to their self-reported scores on the pain subscale of Symptom Checklist-27-plus^43^ using the cut-off score specified in manual. We used this scale because it is a well-validated instrument with a specified clinical threshold that allowed us to differentiate between groups using the cut-off score specified in the manual. The criterion to be included in the sub-clinical pain group was to have a score above the clinical cut-off of 1.77 on the pain subscale based on the manual of Symptom Checklist-27-plus^43^. The cut-off is the official cut-off specified in the manual. As we had used this methodology before in a similar study conducted on university students in Switzerland^11^, we wanted to use the same methodology and data analyses to be able to compare the results. The type of effect size used in our study is Partial eta squared (*η*^*2*^_*p*_), estimated to be 0.027 based on our previous study^11^, which investigated differences in mood and behavioral responses to reward between students with sub-clinical pain symptoms and healthy controls using an ANOVA. We used the recommendations formulated by Lakens^44^ to enter the parameters in G-Power using a partial eta squared (*η*^*2*^_*p*_) of 0.027, which led to the estimated Cohen’s F value of 0.17. Using G-Power, the estimated sample size needed would be 80 to have the actual power with 5% alpha error, 95% power, and p < 0.05 as the significance level for the ANOVA with repeated measures and within-between interaction. Both groups did not differ in depression and anxiety scores (p > 0.05). In addition, the depression mean scores were below the threshold of a score of 11 for significant depression score (mean /SD control: 7.25 ± 3.64; subclinical group: 5.97 ± 3.62).

**Table 1:** Participants’ sociodemographic characteristics and clinical scores (N = 88).

	Sub-clinical Group (N = 40)	Control group (N = 48)	Statistics							
	N (%)	N (%)	Test value	Significance (p)						
**Gender**				0.91						
Female	20 (51.3%)	22(50%)	χ^2^ = 0.14							
Male	19(48.7%)	22(50%)								
**Language**				0.189						
English	35 (89.7%)	30 (75%)	t_(79)=_2.35							
Other	4(10.3%)	10 (25%)								

	Mean (SD)	Mean (SD)								
Age	21.32 (2.31)	22.25(2.33)	t_(81)=_1.81	0.074						

Psychometric measures	Mean	SD	Min	Max	Mean	SD	Min	Max		
HADS (anxiety)	9.45	4.43	0	17	8.66	4.30	0	17	t_(81)=_1.98	0.052
HADS (depression)	7.25	3.64	0	15	5.97	3.62	0	17	t_(81)=_1.96	0.053
SCL-27-plus-socio-phobic symptoms	2.49	1.01	0	4	2.00	1.09	0	4	t_(81)=_1.56	0.122
SCL-27-plus-vegetative symptoms	2.00	0.89	0	3.80	1.99	0.88	0	3.20	t_(81)=_1.78	0.078
SCL-27-plus-agoraphobic symptoms	1.32	0.91	0	3.25	1.00	0.91	0	2.75	t_(81)=_1.51	0.135
SCL-27-plus-depressive symptoms	2.09	1.001	0	4	1.98	1.01	0	4	t_(81)=_1.48	0.142
**SCL-27-plus-pain symptoms**	2.31	0.47	1.33	4	1.00	0.59	0	3	t_(81)_ = 11.05	< 0.01
Headaches	2.87	0.61	2	4	1.70	1.37	0	4		
Chest pains	1.84	1.40	0	4	0.55	1.20	0	4		
Muscle cramps	2.56	0.85	0	4	1.11	1.33	0	4		
Muscle pain/sore muscles	2.76	0.77	0	4	1.09	1.37	0	4		
Pain in arms or legs	1.84	1.11	0	4	0.84	0.99	0	4		
Backaches	1.97	1.03	0	4	0.70	0.85	0	4		
SCL-27-plus-lifetime assessment for depressive symptoms	1.41	0.39	1	2	1.51	0.35	1	2	t_(73)=_-0.85	0.398

This table demonstrates participants’ sociodemographic characteristics and the clinical scores of both groups on different psychometric measures used in the study.

*SCL-27-plus* Symptom Checklist, *HADS* Hospital Anxiety and Depression Scale, *Sub-Clinical Group* participants with clinically significant pain symptoms, *Control Group* participants without clinically significant pain symptoms.

The study was approved by the Institutional Review Board at the University of Fribourg in Switzerland (2017/IRB 334A). Participants were thoroughly informed about the study and the informed consent was obtained from all the participants in our study. All research was performed according to the Declaration of Helsinki. The privacy rights of participants were always observed during our study.

### Procedure

Students completed a battery of questionnaires online using LimeSurvey^®^ (LimeSurvey GmbH, Hamburg, Germany. URL http://www.limesurvey.org) as well as an adapted online version of the Fribourg reward task^10,11^. In addition, the adapted version of the Fribourg reward task was performed online by the participants at their respective homes (due to the COVID situation) without the experimenter. Participants were allowed to terminate the survey at any time. The survey was anonymous, and the confidentiality of information was maintained.

### Psychometric measures

#### Symptom Checklist (SCL-27-plus) for pain

Symptom Checklist (SCL-27-plus^28^) is a multidimensional assessment instrument for mental health status^45^. With 27 items rated on a 5- point Likert-type scale, it consists of five dimensions: depressive, vegetative, agoraphobic, social phobia, and pain symptoms. A lifetime assessment of depressive symptoms and a screening question for suicidality are also included. Participants rated the following pain symptoms: headaches, chest pain, muscle cramps, muscle aches, arm/leg pain, and lower back pain for 0 “*never*” to 4 “*very often*” on a pain subscale depending on how often these symptoms occur in the past 2 weeks. A value of 0 stood for “never”, 1 stood for “1–2 days”, 2 for “3–7 days”, 3 for ”8–12 days”, and 4 for “13–14 days”. A mean score of ≥ 1.77 indicates physical symptoms of pain according to SCL-27^43^. Previous studies reported significant pain symptoms in university students using the SCL-27^11,46,47^. The overall Cronbach’s alpha coefficient in this study was 0.87, which is good.

#### Hospital anxiety and depression scale

Hospital Anxiety and Depression Scale (HADS^48,49^ is a self-assessment scale that consists of a 14-item scale (7 relating to anxiety symptoms and 7 to depression); each item is coded 0 to 3. The total score can range from 0 to 42. The clinical cut-off score on depression or anxiety scales is equal to or greater than 11 on each symptom. The overall Cronbach’s alpha coefficient in this study was 0.80, which is good.

### Fribourg reward task

**Figure 1:**
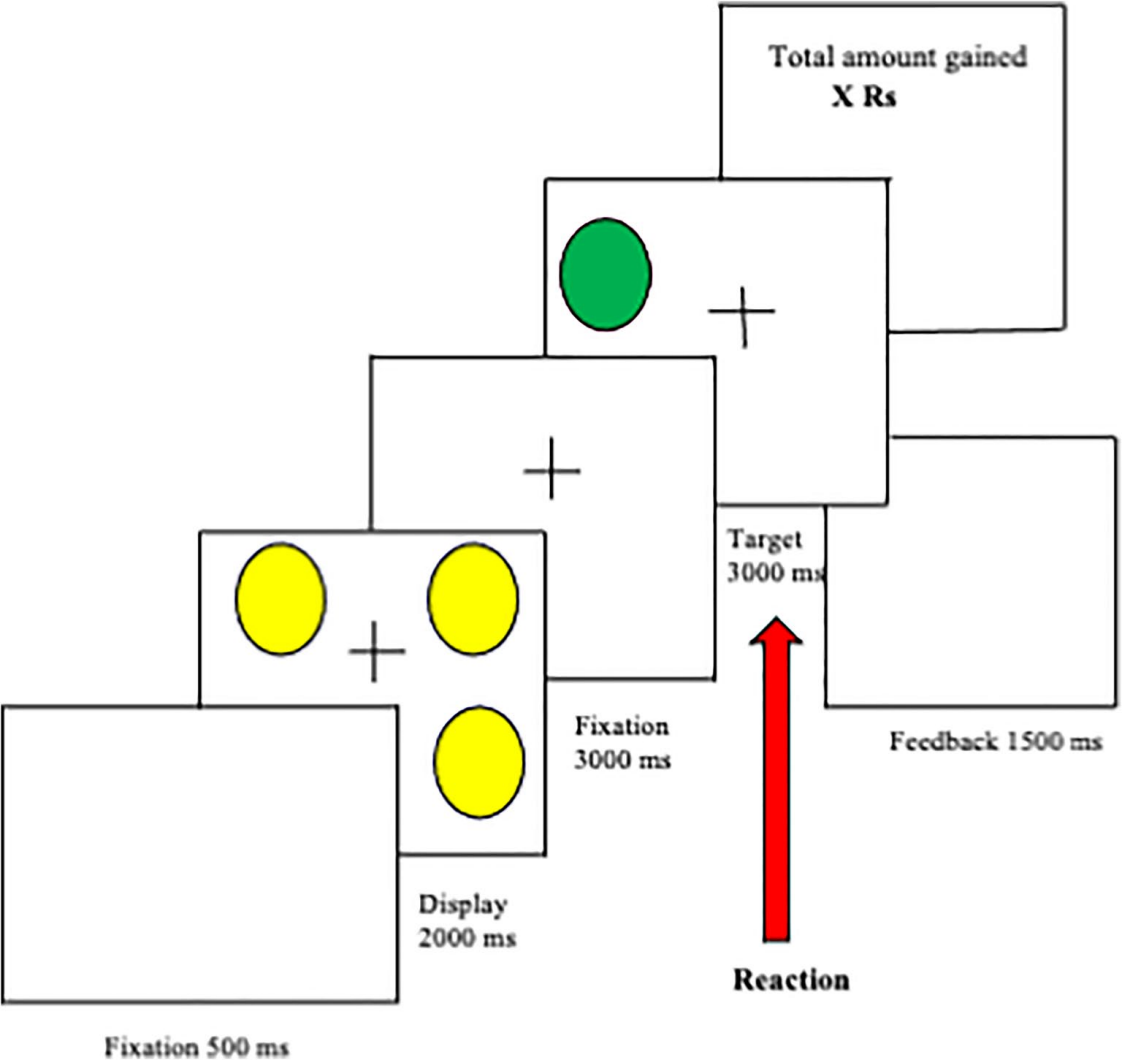
Schematic representation of a trial of the Fribourg Reward Task with 3 circles. This figure shows the schematic representation of a trial of the reward task with 3 circles. In the first display, an array of yellow circles (3 circles) was presented for 2000 ms after a fixation time of 500 ms. After a delay of 3000 ms, a green circle appeared, and the subject had 1500 ms to decide whether the position of the green circle was the same as that of one of the preceding yellow circles. If so, the correct response for participants was to press a button with their right hand. If not, the participants had to press a button with their left hand. After the response time had elapsed, the circle disappeared, and the accumulated amount of money earned appeared on the screen in the reward condition or nothing is shown on the screen in the no-reward condition. During the reward condition, the participants should earn a monetary reward for every correct response.

We used an online adapted behavioral version of the Fribourg reward task^10^ to measure reaction times, and mood reactions to monetary reward. Neuroimaging studies using this task have successfully elicited neural activation in regions associated with the cerebral reward system^50^, including the striatum, a putative region for reward processing. In short, the task was originally programmed using E-Prime software (version 1.1.3, Psychology Software Tools Inc., Pittsburg, Pa., USA) and made available online using OpenSesame, a graphical experiment builder for the social sciences. The experimental task was presented in three block conditions, comprising reward conditions (monetary reward, social reward, and no reward). Here, we focus only on the monetary versus no-reward conditions to investigate whether physical symptoms of pain affect the responses to monetary reward in Indian students as we evidenced in Swiss students^11^. Each block condition consisted of 12 trials and the order of the blocks was pseudo-randomized. In the three-reward conditions, at the onset of each trial (see Fig. 1), a visual cue (2000 ms) was presented (3 yellow circles), along with the reward associated with performance. After the presentation of a fixation cross (500 ms), participants saw an array of yellow circles (3 circles, 2000 ms). A fixation cross (3000 ms) was presented before the visual target. The visual target (a green circle, 3000 ms) was displayed in any position on the screen and signaled that the participant should decide as quickly as possible whether this circle was in the same position as one of the circles presented previously. After response execution and a variable jittered interstimulus interval (ISI; 0 ms or 2000 ms), the feedback screen (1500 ms) informed the participant of their winnings. For the monetary reward condition, a screen with “Rs 0” was shown for incorrect trials or “Rs 10” for correct trials, in the social reward condition, a “neutral” face smiley was shown for incorrect trials or a “win” face smiley for correct trials and in the no-reward condition, a blank screen was shown for every correct or incorrect trial. In the end, a feedback screen (1000 ms) indicating the cumulative amount of monetary reward or social reward (smileys) earned (in the monetary and social reward conditions) or a blank screen in the no-reward condition. Correct responses were associated with monetary gains (“Rs 10” for participants in India) in the monetary reward condition. Correct responses were not associated with any gains in the no-reward condition. We asked participants to rate their momentary mood and stress level using a visual analog scale from 0 (bad mood)—10 (good mood). With smileys at the anchor points (0 = ); (10 = ). Participants rated their momentary mood and stress level on a scale of 0 to 10 at baseline, at the beginning of the experimental session, and before and after each block for a maximal duration of the 20 s. Participants were informed that they would receive the total sum in cash at the end of the session. Participants underwent a training phase before proceeding to the main task. A criterion of 70% correct responses was chosen to prevent arbitrary guessing and thereby verify understanding of the task and ensure that participants would win similar amounts of money.

### Data analysis

Analyses were calculated using IBM SPSS Statistics version 28.0.1 (https://www.ibm.com/spss/statistics). Normality tests were performed for mood and reaction time, and the data were found to be normally distributed using Shapiro–Wilk Test (p > 0.05). Descriptive statistics are presented in Table 1 for both groups. Baseline mood ratings were compared between groups using t-tests. We also performed exploratory analyses comparing the mean mood ratings between groups in the no-reward conditions to test the specificity of our results. To test the effect of reward on mood in participants in the sub-clinical pain group and the control group, a repeated-measures ANOVA was conducted, using mood as the dependent variable, with the following factors: groups (sub-clinical pain group and control group) as a between-group factor, reward conditions (monetary reward and no-reward) as within group factors. Additional mixed ANOVA using the same factors was applied using reaction time as the dependent variable to test the effect of reward on performance and to compare possible performance differences between the two groups of participants.

In addition, we postulated that there would be a significant positive correlation between mood ratings and monetary gains in the reward condition in the control group, but not in the sub-clinical group based on our previous study^11^. To test this hypothesis, the Pearson product-moment correlation between mood scores and monetary wins in reward conditions was performed separately in each group, similar to previous studies^10,11,51^.

### Ethical approval

This study was performed in line with the principles of the Declaration of Helsinki.

Approval was granted by the internal review board (2017/IRB 334A) at the Department of Psychology at the University.

### Informed consent

Participants were thoroughly informed about the study and the informed consent was obtained from all the participants in our study according to the Declaration of Helsinki. The privacy rights of participants were always observed during our study.

## Results

### Reward and mood

Average mood scores in the reward and no-reward conditions are summarized in Table 2. Baseline mood scores were 6.67 ± 1.62 (mean ± SD) for the control group and 6.37 ± 1.51 for the sub-clinical pain group. The results of the t-test showed no significant difference in mood between groups at the baseline (t_54_) = 1.74, p = 0.88), suggesting both groups showed similar mood scores. We also performed exploratory analyses comparing the mean mood ratings between groups and the no-reward conditions and no significant differences were found (t_59_) = 1.72, p = 0.09), suggesting both groups showed similar mood in no-reward conditions. The results of the repeated-measures ANOVA for mood showed no significant interaction effect between group and reward conditions (*F*_*1,59*_ = 0.298, *MSE* = 0.528, *p* = 0.59, *η*^*2*^_*p*_ = 0.001). A significant main effect was found only for the factor of reward (F_1,59_ = 6.127, MSE = 31.88, p = 0.004 significant at 0.01, η^2^p = 0.07). Participants reported higher mood scores in response to reward (M = 7.00, SD = 1.80) compared to no reward (M = 6.58, SD = 2.05). No significant main effect of the groups was seen (F_1,59_ = 0.002, MSE = 0.004, p = 0.96, η^2^p = 0.00).

**Table 2:** Means and standard errors for mood scores in the reward and no reward conditions.

Groups	N	Reward condition Mean ± SE	No-reward condition Means ± SE
Sub-Clinical	40	6.45 ± 0.33	6.50 ± 0.38
Control	48	7.51 ± 0.29	7.39 ± 0.36

The results of the t-test showed no significant difference in mood between groups at the baseline (t _54_) = 1.74, p = 0.88).

This table demonstrates the scores of both the groups. The sub-clinical group showed lower mood scores than the control group.

### Correlations between mood and reward

**Figure 2:**
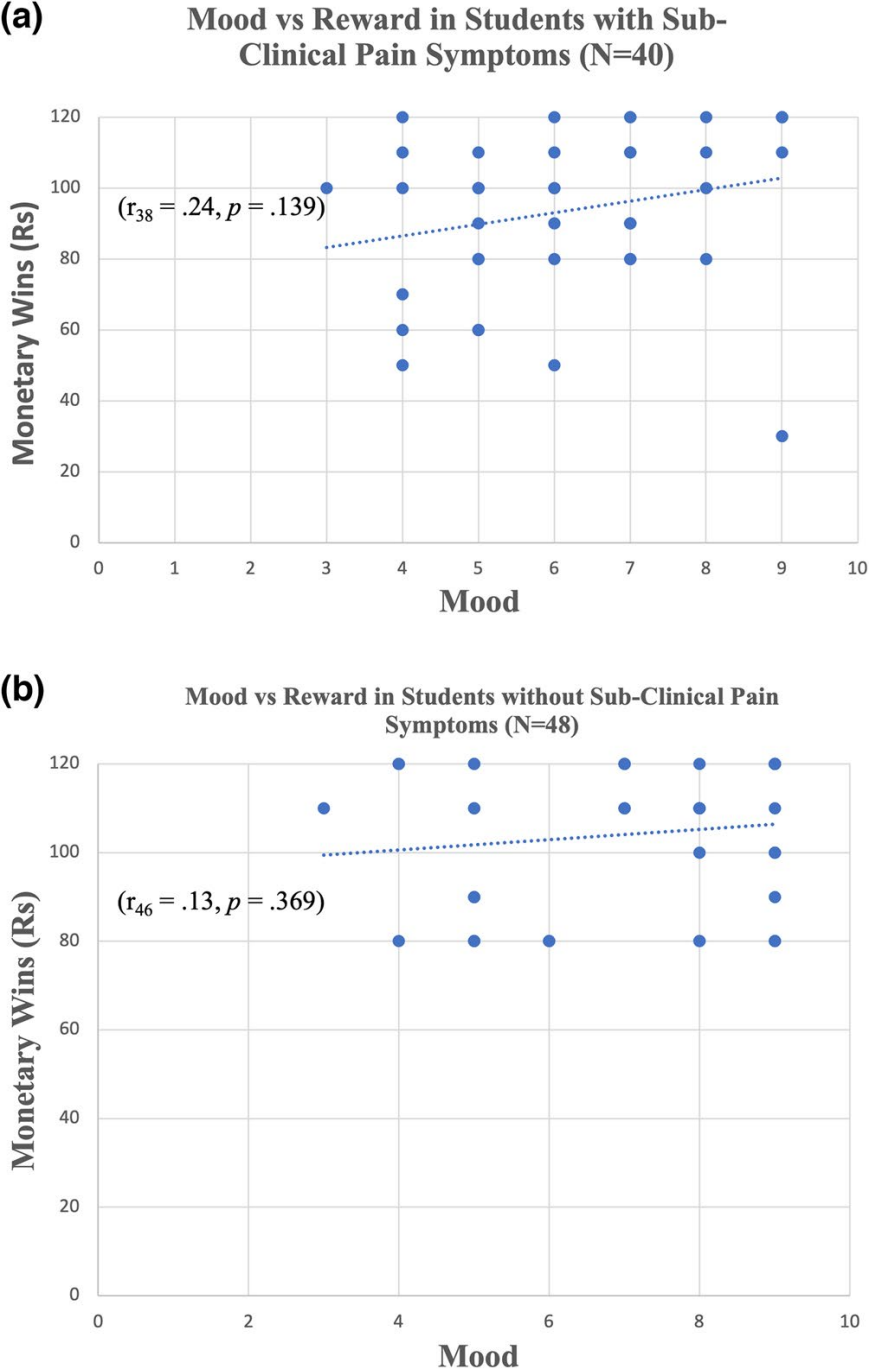
Correlations between the mean mood scores and the amount of monetary reward received. The following different figures demonstrate the correlations between the mean mood scores and the amount of monetary reward. The value of the y-axis shows the sum win (in Rs) obtained by adding the monetary wins in reward conditions. The subjects as represented as dots (blue circle). (**a**) Correlation between mean mood scores and the amount of monetary reward received in the control group without clinically significant pain symptoms of pain (N = 48). The results indicate that there was no significant correlation found (r_46_ = 0.13, *p* = 0.369). (**b**) Correlation between mean mood scores and the amount of monetary reward received in the sub-clinical group with the symptoms of pain (N = 40). The results indicate that there was no significant correlation found (r_38_ = 0.24, *p* = 0.139).

Additional correlations were performed between mood scores and monetary wins. In the control group, no significant correlation was found between mood scores and monetary wins (r_30_ = − 0.085, *p* = 0.646). Also, in the sub-clinical group, no significant correlation was found between mood scores and monetary wins (r_27_ = 0.11,* p* = 0.58) (see Fig. 2). Because non-parametric tests are more sensitive in the case of a non-linear association, we replicated the analyses using a non-parametric Spearman correlation and found that in the control group, no significant correlation was found between mood scores and monetary wins (r_30_ = − 0.069, *p* = 0.709). Also, in the sub-clinical group, no significant correlation was found between mood scores and monetary wins (r_27_ = 0.254,* p* = 0.184).

### Reward and performance

Results for reaction time in the two reward conditions are summarized in Table 3. The mixed ANOVA of reaction time showed no significant effect for the interaction between groups and reward conditions (*F*_*1,63*_ = 0.003, *MSE* = 4160.893, *p* = 0.96, *η*^*2*^_*p*_ = 0.00), A significant main effect was seen for the factor of reward (*F*_*1,63*_ = 4.45, *MSE* = 6,656,851.47, *η*^*2*^_*p*_ = 0.066, *p* = 0.03). Participants were slower in the no-reward condition (M = 7937.25, SD = 6084.73) compared to the reward condition (M = 7470.64, SD = 5572.71). No significant main effect of the groups was seen (*F*_*1,63*_ = 0.198, *MSE* = 14,077,891.91, *p* = 0.66, *η*^*2*^_*p*_ = 0.00).

**Table 3:** Means and standard errors for reaction time (in ms) in the reward and no reward conditions.

Groups	N	Reward condition Mean ± SE	No-reward condition Means ± SE
Sub-clinical	40	7470.65 ± 1034.83	7937.25 ± 1129.91
Control	48	8144.03 ± 1026.31	8587.87 ± 1032.82

Participants reported faster reaction time to reward trials as compared to non-reward trials.

## Discussion

The main aim of the present study was to investigate the prevalence of physical pain symptoms in a sample of university students in India. The second aim was to replicate our previous study conducted to investigate the relationship between monetary reward and pain^11^ in university students in Switzerland in a non-western cultural context i.e., in India. We hypothesized that students reporting physical pain symptoms would show a reduction in the effect of a monetary reward on mood ratings (i.e., reduced mood scores in response to reward) and performance (i.e., higher reaction times in response to reward) compared to the control group. To our knowledge, this is one of the first studies to study the interaction between pain and reward processes in the Indian sample.

Forty-five percent of the university students in India reported high levels of physical symptoms of pain, which are in line with our previous study conducted on a sample of students in Switzerland^11,47^. In general, studies show that students spend approximately 5 h/day in a sitting position and prolonged sedentary behavior adds up to this problem^52,53^. Specifically, in India, certain fields of education demand long working hours from students. For example, in the field of medicine, many competitive exams are conducted for undergraduate and postgraduate medical courses which are difficult to pass and lead to a lack of physical activity, stress, and excess use of laptops and phones^54^. This makes some students more prone to developing musculoskeletal pain-related issues^55^.

Regarding our hypothesis that the effect of reward on mood and performance would be reduced in participants with sub-clinical pain, our ANOVA results did not show any significant interaction between reward and groups neither for mood scores nor for the outcomes related to performance, i.e., reaction times. Even though, an effect was seen for reward (i.e., independently of pain status, participants’ mood and faster reaction time to reward was higher compared to non-reward trials). Interestingly, the time taken by the participants to respond to the stimuli in our study was longer than 8000 ms. However, this is still an intriguing result, because the results obtained in a group of Swiss students showed mean reaction times between 1500 and 4000 ms and a previous study using this task also obtained average reaction times between 2500 and 3000 ms^51^. This could be related to the fact that no maximal reaction times were fixed in the version of the task used for this study. In addition, the task was performed online (due to the COVID situation) without the experimenter while in previous studies the experiment was performed in the lab with the presence of an investigator.

Our results did not show a significant correlation between mood ratings and monetary wins. Interestingly, this is not in line with our previous study conducted on university students in Switzerland^11^. In many previous studies which were performed on WEIRD samples, it is seen that pain alters the motivation to obtain reward and leads to reduced mood responses^56,57^. However, we did not see this effect of pain on reward processes in the Indian students, although pain symptoms were reported in our sample. This might be explained as the experience of pain and pain-related impairments differ across cultures^33,58,59^. For instance, one of the studies on chronic pain conducted in India and the US showed that people in India endorsed high pain tolerance and less frequently experienced pain-related impairments as compared to their counterparts in the US^60^. On one hand, this could be explained as individuals in South Asian countries are encouraged to avoid pain due to a lack of financial support to seek a medical doctor as compared to western countries^32,33^ and on the other hand, this could also be explained as India is a collectivistic society^61^ having strong family ties and friendship groups and many previous studies have shown that social support which is one of the qualities of the collectivistic culture might act as a protective factor, leading to lower levels of high pain experience in people with chronic pain^62,63^. Secondly, pain-related impairments are often augmented by psychopathological problems^64^. For example, depression is ranked as one of the strongest predictors of back pain^65^. According to Marbach and Lund^66^ and Garland, Trøstheim^67^, one of the reasons for blunted responses to monetary reward might be due to decreased interest and pleasure in response to positive stimuli (i.e. anhedonia) which is one of the key features of people suffering from depression and from a clinical standpoint, high rate of comorbidity between pain and depression has been seen in the previous studies^12,68^. One of the studies conducted on university students in North America (N = 618) showed that students higher with psychopathological problems showed a higher prevalence of chronic pain^69^. In our sample, however, both groups did not differ with regard to depression scores and their mean scores were below the clinical threshold for clinically significant depressive symptoms. This might indicate that our sub-clinical population did not show pain-related impairment due to lower symptoms of psychopathological problems. However, there is a stigma related to mental health (at least to consulting a mental health specialist) in India^70,71^ and this might have biased the self-reported depression ratings. Finally, it is important to address that the cut-off for significant pain was the one available from Symptom Checklist-27-plus^43^, and might not represent the reality of the Indian population. This aspect remains to be investigated in future studies.

Taken together, contrary to our previous findings in the Swiss students^11^, pain-related impairment on monetary-reward processes was not observed in this sub-clinical Indian sample. This shows that pain-related impairment may not be a universal phenomenon, and it can vary across cultures^72^. Furthermore, it may show that different levels of pain are needed across cultures to reflect an impact on reward processing. Most of the previous studies related to pain were carried out in Western samples^73^, and, to our knowledge, none of the studies have investigated the relationship between reward pain from non-Western specifically from an Indian perspective.

The absence of replication of our findings with Swiss students in our Indian sample highlights the importance of investigating non-WEIRD populations, as there is an underrepresentation of studies focusing on non-WEIRD samples. For instance, people might tend to view pain-related impairments in reward processes as a universal human phenomenon, while this process might be more specific to some cultures than others. Therefore, our study conducted on Indian students fills this gap and provides insight into the fact that the blunted association between mood and reward in a sub-clinical population i.e., impaired responses to monetary reward might not be universal.

Some limitations merit attention. First, the measurement of pain based only on the Symptom Checklist-27-plus^43^ is a limitation of the present study and the use of self-report instruments can lead to memory bias and greater subjectivity in the responses, in particular for questions related to stigma such as those related to depression. However, self-report is potentially the best way to obtain responses related to mood and pain. Also, the length of our online questionnaires may have led to less accurate answers due to fatigue, even though participants could take breaks. Second, transforming a variable from continuous (i.e., self-reported pain scores) to categorical (i.e., control group vs sub-clinical pain group**)** might have reduced the statistical power. In that context, using linear model analyses could have been better, considering the variability of our data. However, we chose a data analysis strategy that allowed for comparison with our previous study performed in a Swiss sample. Third, there was no cut-off given in the Symptom Checklist-27-plus^43^ manual for the Indian population, the cut-off used in our study, was for a European population, which might not be representative of the Indian reality. Future studies should seek to validate this instrument in Indian and other non-WEIRD samples as well as determine specific cut-offs for these populations. Fourth, our data only comprised undergraduate students which limits the interpretation of our results to this population. Fifth, there was a trend seen in the p-value of HADS (Depression) to be statistically significant, however, the depression mean scores were below the threshold of a score of 11 for a significant depression score.

In conclusion, our findings provide very promising evidence of the cultural variations between pain and reward processes. Our study is the first to study the relationship between pain and reward in university students in India. This relationship in a sub-clinical population provides the first insight into the development of culturally specific preventive interventions. This study also highlights that students all around the world are suffering from high symptoms of pain and more research is needed to explore the association between psychopathological problems and physical pain.

## Data Availability

The datasets analyzed during the current study are deposited in the Dataverse repository (10.7910/DVN/8CM4Y5).
